# The Effect of Frailty Syndrome on the Quality of Life of Individuals with Parkinson’s Disease: A Pilot Observational and Multicenter Study on the Polish Population

**DOI:** 10.3390/ijerph192215226

**Published:** 2022-11-18

**Authors:** Aleksandra Pytel, Jan Aleksander Beszlej, Monika Biercewicz, Anna Roszmann, Dorota Krówczyńska, Aleksandra Kołtuniuk

**Affiliations:** 1 Department of Nursing and Obstetrics, Faculty of Health Science, Wroclaw Medical University, 51-618 Wroclaw, Poland; 2 Department of Psychiatry, Wroclaw Medical University, 50-367 Wroclaw, Poland; 3 Clinic of Geriatrics, Faculty of Health Science, Collegium Medicum in Bydgoszcz, Nicolaus Copernicus University in Toruń, 85-094 Toruń, Poland; 4 Division of Neurological and Psychiatric Nursing, Medical University of Gdańsk, 80-211 Gdańsk, Poland; 5 Cardinal Stefan Wyszynski Institute of Cardiology, 04-628 Warsaw, Poland; 6 Department of Nursing and Obstetrics, Collegium Mazovia, 08-110 Siedlce, Poland

**Keywords:** Parkinson’s disease, frailty syndrome, quality of life, depression, daily activity

## Abstract

Parkinson’s disease (PD) is a neurodegenerative disorder involving decreased dopamine release and atrophy of dopaminergic neurons of the substantia nigra. Frailty syndrome (FS) is common in older adults, which, in combination with PD symptoms, can substantially affect the quality of life (QOL). This study aimed to assess the prevalence of FS among PD patients and to identify variables affecting their QOL with particular attention to FS. The study included 296 patients (*n* = 173 women) with a mean age of 70.3 ± 5.7 years suffering from PD for an average of 8.2 ± 5.6 years. Patients were classified as at least stage II according to the Hoehn and Yahr scale. The following standardized questionnaires were used in the study: Schwab and England Activities of Daily Living (SE-ADL), Parkinson’s Disease Questionnaire (PDQ-39), Beck Depression Inventory (BDI), Unified Parkinson’s Disease Rating Scale (UPDRS), and Tilburg Frailty Indicator (TFI). FS was found in 96% (*n* = 283) of the PD patients studied. No depression occurred in 30% (*n* = 89) of subjects, moderate depression in 48% (*n* = 141) of subjects, and severe depression in 22% (*n* = 66) of subjects. The mean score of the PDQ-39 questionnaire in PD subjects with FS was 41.6 pts (min–max: 5.2–81.5 pts; SD = 17.4 pts), which was statistically significantly higher than in subjects without FS (*p* < 0.05). FS has been shown to be present in most of the subjects with PD. FS occurs more frequently with a longer PD period, which is associated with reduced physical capacity and QOL. Physical activity improves QOL and reduces disease progression. FS, similar to PD, is a common cause of disability in older adults and their dependency. Predictors such as depression, advanced stage of the disease, higher education, and low professional and economic status significantly affect the QOL level of PD patients. However, the results obtained among the Polish population of PD patients do not confirm the impact of FS on the QOL, so there is a need to conduct further research on this subject.

## 1. Introduction

Parkinson’s disease (PD) is the second most common neurodegenerative disorder after Alzheimer’s disease, and the incidence is expected to continue rising as the global population ages. Degeneration of dopaminergic neurons of the substantia nigra of the midbrain with their atrophy plays a key role in the etiopathogenesis leading to a drastic dopamine deficiency throughout the striatum [1]. Dopamine deficiency causes typical symptoms such as resting tremors, muscle rigidity, bradykinesia, hypokinesia, abnormal postural reflexes, poor posture, and freezing symptoms. Additional symptoms include the presence of hyposmia, gastrointestinal disorders, mental dysfunction (depression, anxiety, psychosis), sleep disorders, or dysphagia [2].

More than 1 million PD patients reside in Europe and there are approximately 5 million worldwide. Although no accurate epidemiological studies have been conducted in Poland, it can be assumed that 60–80 thousand Polish people have been diagnosed with PD. Given the growing population of older adults in Europe, this number is expected to double by 2030, posing a serious challenge to the entire healthcare system [3]. Reports by Dorsey et al. [4] for 2005–2030 and Bach et al. [5] for 2010–2030 indicate that chronic diseases, including PD, will be both a medical problem and a social problem. Both reports show that as the global population ages, the number of people with chronic diseases, including PD, will increase, and the number of PD cases will double by 2030 (from 4.1–4.6 million to 8.7–9.3 million in 2030). Population aging is one of the most significant demographic and social trends of the 21st century. In 2021, more than one-fifth of the European Union (EU) population was 65 years of age or older, and this segment is projected to grow to 31.1% by 2100 [6].

In recent years, also in Poland, there has been increasing population aging. As a result, the share of older adults in the population of Polish residents is steadily increasing. At the end of 2020, the number of people aged 60 and over reached 9.8 million, increasing by 1.0% compared to the previous year. The percentage of Polish older adults reached 25.6%. According to a forecast by the Central Statistical Office (Polish: Główny Urząd Statystyczny, GUS), the population of people aged 60 and over in Poland is expected to rise to 10.8 million in 2030 and reach 13.7 million in 2050. These people will comprise approximately 40% of the Polish population [7].

According to data from the National Health Fund (Polish: Narodowy Fundusz Zdrowia, NFZ), the financial resources allocated for geriatric services reimbursement within the framework of specialized outpatient care and inpatient treatment in 2020 amounted to PLN 88.7 million, of which 96.6% of this amount was allocated to inpatient geriatric care. In Poland in 2020, 67,000 residents of stationary social welfare institutions were people over 60 years of age. Most of them (25,357 people, 24.1%) stayed in social welfare homes [8].

However, an epidemiological study conducted in long-term care facilities in six European countries showed that Poland had one of the highest percentages of residents with poor functional and cognitive status [9].

Therefore, promoting physical activity among older people to maintain their satisfactory health, physical activity, functional fitness, and self-reliance has become one of the prioritized strategic areas established by the WHO for European countries during the period 2016–2025. In the face of such a demographic situation, it becomes necessary to focus social policy on extending life and taking action to improve the QOL of older adults. Additionally, the population of PD patients both in Poland and worldwide should be included. Actions should be focused on identifying the most important determinants of PD, such as stage of disease, FS, depression, and sociodemographic features [10].

According to Singleton et al. [11], PD undoubtedly affects the quality of life (QOL) due to the presence of a multiplicity of symptoms. Therefore, it becomes a priority to continuously analyze the QOL level of this group of patients to properly allocate financial resources to provide optimal care for PD patients and continuously search for deficit areas that require improvement in the level of care provided. According to Villani et al. [12], despite the growing interest in frailty syndrome (FS), there are no well-designed studies on the effect of FS on the QOL of people with PD. This issue remains poorly understood to date.

Many researchers [5,13] have shown the undeniable impact of PD on the lowering of the QOL level of PD patients resulting mainly from the presence of multiple symptoms. According to Keränen et al. [14] and Boland et al. [15], the severity of PD is associated with increased costs and decreased health-related QOL (HRQoL). Slowing the progression of the disease through the use of effective drug therapies in combination with effective and safe therapeutic interventions can result in lower healthcare demands, which in turn can result in reduced costs and improved QOL levels for patients. Therefore, according to Boland et al. [15] and Dodel et al. [16], it becomes most important to analyze the QOL level of PD patients constantly. Only proper management of health policies enables the delivery of patient care at the highest level. Furthermore, it enables the search for effective solutions to minimize the risk of depression and anxiety in the overall therapeutic process.

The literature describes two models of FS, the phenotype model and an accumulation model of deficits of the reduced capacity of systems and systems. Fried et al. [17] describe the phenotype model of FS as the occurrence of exhaustion, a decrease in gait speed, a feeling of lack of strength in the hands, and unintentional weight loss. The second model reports FS as an adverse effect of multiple components such as emotional disturbances, decreased muscle mass, reduced exercise tolerance, motor slowing, incontinence, decreased appetite, and cognitive decline. According to the American Geriatrics Society, FS is a physiological condition of the aging organism due to a decrease in resistance to stressors and a reduction in physiological reserve combined with a dysfunction of the immune and endocrine systems. In 2013, a consensus of six international societies defined SUS as “A multifactorial clinical syndrome characterized by decreased strength, endurance, and reduction in physiological processes, increasing an individual’s vulnerability to the development of dependence on others and/or death” [18].

FS should be considered in a multifaceted way. It implies a reduction in physiological reserves, dysfunction of many organs, and a breakdown in homeostasis, affecting physical, psychological, and social functioning [10,11].

It should be noted that cognitive impairment in the course of FS is different from physiological brain aging. FS, like PD, is a common cause of disability in older adults and causes dependency. Risk factors for developing FS, such as advanced age, depressed mood, and gait disturbances, often coexist with the course of PD. This suggests that people with PD are predisposed to FS [19,20].

This study aimed to assess the prevalence of FS among PD patients and to identify variables affecting QOL with special attention to FS. It may be clinically relevant to modify, prevent, or treat such factors and improve the QOL of PD patients.

## 2. Materials and Methods

### 2.1. Study Design and Characteristics of the Study Group

The study was conducted between March 2017 and March 2021 among patients belonging to the Association of Parkinson’s Disease Patients and Their Families based in Gdynia, Krakow, the Polish Society for Combating Disability—Circle of Friends of People with Parkinson’s Disease in Wroclaw, Regional Association of People with Parkinson’s Disease in Walbrzych, patients of the “PROVITA” Specialized Preventive and Therapeutic Facility in Wroclaw, Leszno Association of People with Alzheimer’s and Parkinson’s Disease in Leszno, and the Mazovian Association of People with Parkinson’s Disease based in Warsaw.

Patients who met the inclusion criteria responded to traditional self-administered pencil-and-paper questionnaires, which were designed to be completed in approximately 20 min. The group of study respondents was recruited during monthly meetings with PD patients and while waiting for medical appointments and physiotherapy sessions.

The study included 348 PD patients, of which 52 did not complete the questionnaires successfully. Finally, 296 completed questionnaires from PD patients were obtained in the following cities: Wroclaw (*n* = 91), Warsaw (*n* = 79), Gdynia (*n* = 29), Krakow (*n* = 40), Leszno (*n* = 28), and Walbrzych (*n* = 29).

Subjects with a PD diagnosis based on diagnostic criteria according to the United Kingdom Parkinson’s Disease Society Brain Bank (UK PDS BB), at least stage II according to the Hoehn and Yahr scale, and aged 65 years or older were included in the study. The subjects did not have any other neurodegenerative comorbidities, which was also an inclusion criterion. The study involved individuals who provided informed consent to participate in the study after familiarizing themselves with the objectives and procedure of the study. One set of anonymous questionnaires was allocated to each respondent. After completing them, each was measured for handshake strength using a dynamometer. The present study was approved by the Bioethics Committee of the Wroclaw Medical University (permission no.: KB-534/2016).

### 2.2. Research Tools

The study was conducted using standardized questionnaires: Schwab and England Activities of Daily Living (SE-ADL), Parkinson’s Disease Questionnaire (PDQ-39), Beck Depression Inventory (BDI), Unified Parkinson’s Disease Rating Scale (UPDRS), Tilburg Frailty Indicator (TFI), and a self-constructed questionnaire assessing sociodemographic and clinical variables.

#### 2.2.1. Schwab and England Activities of Daily Living (SE-ADL)

The (SE-ADL) includes 11 descriptions of the patient’s condition, namely: (1) 100%—I am completely independent of my surroundings, I perform all daily activities without difficulty or slowing down, normal mobility, no sense of inability; (2) 90%—I am completely independent of my surroundings, I am able to perform all daily activities with some slowing down and difficulty, I need 2 times as much time for some activities as before the disease, I am aware of my mobility difficulties; (3) 80%—I am completely independent in most activities, I need 2 times as much time for most activities, I am aware of my difficulty in moving and slowing down; (4) 70%—I need help with some activities, I am 3–4 times slower at some activities, I spend most of my day doing basic activities; (5) 60%—I am partially dependent on my surroundings, I do some activities on my own, but very slowly and with a lot of effort, some activities I am unable to do; (6) 50%—I am more dependent on my surroundings, I need help with half of my daily activities, I am slower, I have difficulty with everything; (7) 40%—I am very dependent on my surroundings, I need help with all activities, only some activities I do on my own; (8) 30%—very few activities I can try to perform on my own at the expense of great effort, I can only start them, further I need help; (9) 20%—the patient is unable to perform anything on his own, he helps with some activities; (10) 10%—the patient is completely dependent on the environment, he cannot even help with activities, total disability; (11) 0%—the patient is immobilized in bed, some vegetative activities are disturbed (swallowing, urination, and bowel movements). The patient marked the description that best described their condition and abilities. The higher the percentage score, the better the patient’s functional ability. The α-Cronbach coefficient for the SE-ADL is 0.85–0.92 [21,22].

#### 2.2.2. Parkinson’s Disease Questionnaire (PDQ-39)

The PDQ-39 was used to assess QOL, describing 39 difficulties the patient may have experienced in daily life and indicating with what frequency they occur (a five-point scale from “never”—0 pts to “always”—4 pts). The questionnaire assesses overall QOL and QOL in 8 specific domains: mobility (10 items), activities of daily living (6 items), emotional well-being (6 items), stigma (4 items), social support (3 items), cognitions (4 items), communication (3 items), and bodily discomfort (3 items). QOL in each domain is expressed by a number ranging from 0–100. Higher numbers indicate a worse level of QOL. There are no standardized values for the PDQ-39, so there is no indication of whether respondents’ scores represent high or low QOL. It can only compare domains among themselves to identify areas of best and weakest QOL. The α-Cronbach coefficient for the PDQ-39 questionnaire is 0.59–0.94 [23,24].

#### 2.2.3. Beck Depression Inventory (BDI)

The BDI, consisting of 21 items on depressive symptoms, was used to assess the patient’s mental state. Each heading has four response options scored consecutively from 0 to 3 points. They are graded descriptions of the severity of depressive symptoms (assessed in the time perspective of the past two weeks). The patient selects the statement that describes their mental state most accurately. A higher number of points indicates more severe depressive symptoms, namely 0–9 pts: no depressive symptoms, 10–19 pts: mild depressive symptoms, 20–25 pts: moderate depressive symptoms, and more than 25 pts: deep depressive symptoms. The α-Cronbach coefficient for the BDI questionnaire is 0.93–0.95 [25].

#### 2.2.4. Unified Parkinson’s Disease Rating Scale (UPDRS)

The UPDRS was used to assess the clinical status of a person with PD and their QOL. In assessing activities of daily living and motor function, UPDRS allows for evaluating the overall QOL of patients with PD and their QOL in 4 specific domains: intellectual functioning, activities of daily living, motor skills, and complications of treatment. The scale contains 42 statements, with the patient marking the severity of a given symptom on a scale from 0 to 4 pts. There are no norms for the UPDRS, so there is no indication of whether respondents’ scores indicate high or low QOL. In addition, in each domain, the scores belong to a different range, which makes it impossible to compare domains with each other; however, higher scores indicate worse QOL. The α-Cronbach coefficient is 0.96, while the coefficient for the Polish version developed in 2021 is 0.90 [26,27].

#### 2.2.5. Tilburg Frailty Indicator (TFI)

The TFI developed by Gobbens et al. [28,29] is the only standardized survey tool adapted to Polish conditions that globally and reliably examines FS. Thus, in order to treat FS as an early preclinical condition, a tool is required that selects those who are functional but at risk of developing a disability. The TFI identifies all three dimensions of human existence, making it possible to recognize multidimensional FS both in an individual person and at the population level. It is based on the assessment of physical, psychological, and social exponents of functioning. The TFI consists of 2 parts: part A includes 10 questions on the determinants of FS (e.g., age, sex, marital status, education level, lifestyle, the presence of chronic diseases, traumatic events in the last year, and living environment), and part B consists of 15 questions ranked according to 3 different dimensions: physical dimension (0–8 points) is assessed by 8 questions on physical health, i.e., unintentional weight loss, difficulty walking, problems with balance, hearing, vision, hand strength, and physical fatigue; mental dimension (0–4 points) includes 4 questions on cognitive function, depression, feelings of anxiety and coping; the social dimension (0–3 points) includes 3 questions related to loneliness, social relationships, and social support. The TFI score ranges from 0 to 15, while FS is diagnosed with a score of at least 5 points. The TFI has a high ability to detect multidimensional deficits, making it a suitable method for FS testing for preventive purposes. The TFI is valid and reproducible for the assessment of frailty syndrome among the Polish population. Cronbach’s alpha reliability coefficients of the instrument range from 0.68 to 0.72 [29].

### 2.3. Statistical Analysis

Statistical analysis was performed using Statistica 13.1 software (TIBCO Inc., Palo Alto, CA, USA). The sample size analysis was performed on the basis of the most important primary objective, based on preliminary studies (unpublished, *n* = 30) in which the difference in QOL (PDQ-39) was compared with the prevalence of FS. The sample size estimation analysis used means and standard deviations of PDQ-39 in both FS and non-FS groups. The estimated sample size is based on a two-sample *t*-test (independent-sample *t*-test). The alpha level was set at 0.05 and the test power at 0.8. It was also assumed that the assessed variables were not correlated, and a two-sided null hypothesis was adopted. Based on the parameters, an estimated sample size of 247 people in the study was obtained. Additionally, it was assumed that the sample size would be 20% higher due to the risk of not obtaining all correct data. The final estimated sample size was set at 296 people. Arithmetic means, medians, standard deviations, and range of variation (extreme values) were calculated for measurable variables. The frequencies of occurrence (percentages) were calculated for qualitative variables. Determination of differences between groups was carried out using the chi-square test. All quantitative variables were checked with the Shapiro–Wilk test to determine the type of distribution. The differences between groups were determined using the non-parametric Mann–Whitney U test or Kruskal–Wallis ANOVA and post hoc analysis (Dunn’s test). Spearman’s rank correlation analysis between selected variables was also performed. Additionally, an analysis of the influence of selected factors on the PDQ-39 score was performed using linear regression. The next step was to build a multivariate model. The model-building process was carried out using stepwise backward regression. All comparisons were taken at the α = 0.05 level.

## 3. Results

Table 1 presents detailed information regarding the study group, which consisted of 296 PD patients. The average age was 70.3 ± 5.7 years, and the average duration of the disease was 8.2 ± 5.6 years. Most subjects were females (58%; *n* = 173) and individuals from a city of more than 100,000 inhabitants (58%; *n* = 172). Among the respondents, most had a university education (36%; *n* = 105), were married (64%; *n* = 189), and were retired (59%; *n* = 174). More than half of the patients (*n* = 158) rated their financial status as good, and 50% (*n* = 149) ran a household with their family. The majority performed additional physical activity during the day or week (77%; *n* = 227) and improved their knowledge of PD (81%; *n* = 239). Most respondents (31%; *n* = 92) said that the disease had not affected the number of new acquaintances or worsened relationships with current ones.

Table 2 presents information on PD and comorbidities. In 56% (*n* = 167) of the subjects, PD occurred in a combined form, in 30% (*n* = 88) in an akinetic-hypertonic form, and in 14% (*n* = 41) in a tremor form. In the majority (56%; *n* = 166), PD symptoms appeared on the right side of the body. The most common comorbidities in the subjects were osteoarthritis (55%; *n* = 163) and hypertension (50%; *n* = 147), and the least common were renal failure (3%; *n* = 9) and stroke (4%; *n* = 12).

Table 3 shows the questionnaire scores of the study group. The mean PDQ-39 total score was 41.2 pts (min–max: 5.2–81.5 pts; SD = 17.7). In the individual domains, the highest mean score was obtained in the “Bodily discomfort” domain with 52.0 pts (min–max: 0.0–100.0 pts; SD = 22.2 pts), and the lowest in the “Social support” domain with 27.1 pts (min–max: 0.0–100.0 pts; SD = 24.7 pts). The mean TFI total score was 9.2 pts (min–max: 3.0–15.0 pts; SD = 1.9). The mean SE-ADL total score was 72.9% (min–max: 20.0–100.0%; SD = 17.7%). The mean BDI total score was 17.3 pts (min–max: 1.0–47.0 pts; SD = 10.8). In the individual domains of UPDRS, the highest mean score was obtained in the “Motor skill” domain with 22.3 pts (min–max: 3.0–52.0 pts; SD = 12.3 pts), and the lowest in the “Intellectual functioning” domain with 4.3 pts (min–max: 0.0–14.0 pts; SD = 3.2 pts). The mean PDQ-39 total score was 41.2 pts (min–max: 5.2–81.5 pts; SD = 17.7). In the individual domains, the highest mean score was obtained in the “Bodily discomfort” domain with 52.0 pts (min–max: 0.0–100.0 pts; SD = 22.2 pts), and the lowest in the “Social support” domain with 27.1 pts (min–max: 0.0–100.0 pts; SD = 24.7 pts).

Table 4 shows a comparison of PDQ-39 scores depending on the TFI category and BDI category. The mean PDQ-39 questionnaire score of patients with FS was 41.6 ± 17.4 pts and was statistically significantly (*p* < 0.05) higher than that of patients without FS. The mean PDQ-39 score in those without depression was 33.0 ± 16.8 pts, in those with moderate depression, it was 41.0 ± 15.4 pts, and in those with severe depression, it was 52.6 ± 17.5 pts. The results were statistically significantly different (main effect *p* < 0.05), while the post hoc analysis showed statistically significant differences between the scores of those without depression, those with moderate and severe depression, and those with moderate and severe depression (*p* < 0.05).

Table 5 shows the comparison of gender, marital status, occupational activity, material status, and household management depending on the TFI category. The majority of those with FS were women (60%; *n* = 171), while men outnumbered those without FS (85%; *n* = 11). The results were statistically significantly different (*p* < 0.05). Otherwise, there were no statistically significant differences (*p* > 0.05).

Table 6 presents the assessment of the impact of selected parameters on the level of the PDQ-39 total score (single-factor model of the predictors included in the analysis). The non-standardized and standardized regression coefficient, standard error, and level of statistical significance were determined. The following variables were included in the analysis: age [years], PD period [years], SE-ADL, BDI, UPDRS, TFI, sex (ref. female), place of residence (ref. village, education (ref. primary), marital status (ref. widowed), occupational activity (ref. pension), material status (ref. very bad), BDI (ref. no depression), and TFI (ref. no FS).

Linear regression analysis in the univariate model showed the influence of PD period (B = 0.73, *p* < 0.001), SE-ADL (B = −11.75, *p* = 0.044), UPDRS: intellectual functioning (B = 1.93, *p* < 0.001), activities of daily living (B = 0.83, *p* < 0.001), motor skills (B = 0.57, *p* < 0.001), complications of treatment (B = 1.48, *p* < 0.001), higher education (B = −6.29, *p* < 0.001), unemployed (ref. pension, B = −6.29, *p* < 0.001), material status (ref very bad, bad: B = 5.59, *p* = 0.032, good: B = −7.97, *p* < 0.001, very good: B = −7.83, *p* = 0.033), BDI (ref. no depression; severe depression B = 10.4, *p* < 0.001) on quality of life PDQ−39. Linear regression analysis in a multivariate model (backward stepwise) confirmed the effect of PD period (B = 0.41, *p* = 0.013), BDI (ref. no depression; severe depression B = 6.2, *p* < 0.001), SE-ADL (B = −12.2, *p* = 0.015), and activities of daily living (B = 0.58, *p* < 0.001).

## 4. Discussion

The contents of the latest National Census of Population and Housing, published by the GUS in 2022, show that Polish society is aging. At the turn of the decade, the percentage of people in the pre-working age group changed from 18.7% to 18.4%, in the working age group from 64.4% to 59.3%, and in the post-working age group from 16.9% to 22.3%. Currently, one in five Polish residents is older than 60. In view of the above, there is a need for research on the QOL of older adults and the identification of factors that may reduce the QOL level of the Polish elderly population, including those suffering from PD [7].

PD is chronic and progressive in nature, so the level of QOL is determined by many factors. The results of the PDQ-39 questionnaire, as in Gozdek et al. [24], showed that PD causes patients to experience great physical discomfort and impairs their mobility and mental and emotional functioning. In our study, the mean main score (PDQ-39—total) was 41.2 ± 17.7 pts. In the individual domains, the highest mean score was obtained in the “Bodily discomfort” domain, 52.0 ± 22.2 pts, and the lowest in the “Social support” domain, 27.1 ± 24.7 pts. The apparent lack of social support among the study group is an important exponent in the identification of factors affecting the QOL of PD patients and in the planning of health policy in Poland and worldwide and is also confirmed in the above studies.

Winter et al. [30] showed that age is a significant variable affecting the QOL of PD patients among sociodemographic factors. However, our study did not confirm this relationship. Similar results were obtained in studies by Ziropada et al. [31], Gozdek et al. [24], and Yamanishi et al. [32], which also failed to confirm a correlation between PD patients’ age and their QOL. In our study, as in the studies cited above, lower material status, inactivity, higher education of PD patients, and disease duration were found to be important determinants of QOL.

Lorencowicz et al. [33] indicate a solid relationship between functional fitness and the level of QOL in five of the nine domains of the PDQ-39 questionnaire. Our study showed the same relationship: the higher the patient’s fitness, the better the QOL (*p* < 0.05).

According to Brinkman et al. [34] and Rafferty et al. [35], exercise rehabilitation is an effective form of non-pharmacological management of PD. In addition, the literature points to its significant role in the treatment process due to its beneficial effects on aggravating symptoms of the disease. Similarly, our study showed that physically active patients had higher fitness levels than those who did not engage in any activity.

Another important determinant of QOL is the functional status of PD patients. Similar to the study by Gliwicz et al. [36] and He et al. [37], the level of QOL depended on the PD stage. The more advanced the stage of the disease, the lower the level of QOL (β = −0.12; *p* = 0.044).

QOL in PD patients is determined not only by the severity of the disease and associated dysfunction in daily living activities but immensely by neuropsychiatric disorders in the form of depression.

Lorencowicz et al. [33] showed depression of varying degrees of severity in as many as 64% of the subjects. In a study by Rosińczuk et al. [38], 48% of subjects had moderate depression, and 8% had severe depression. It is important to note that depression can worsen QOL more than disease progression. Similar to the study of Gozdek et al. [24], the present study confirms that the severity of depressive symptoms significantly affects all nine domains of QOL; the greater the severity of depressive symptoms, the worse the QOL.

The fact is that FS is a common phenomenon in older persons. The prevalence of frailty ranges between 4% and 59% in older adults and is higher in women than in men. More than 50% of the European population aged >50 years are pre-frail or frail (the overall prevalence of pre-frailty was 42.9% and frailty was 7.7%). The prevalence of frailty in Europe is estimated at approximately 3–15.6%, and in Poland, it is 3.1% [39]. The studies show that the prevalence of frailty and pre-FS is of higher incidence in inhabitants of formal long-term care (LTC) facilities than in people living in the community [40].

According to Sokolowski et al. [41], current knowledge regarding FS and in people living with PD is limited. There is no widely used screening tool for FS in PD, and more research is required to determine the best method to identify this syndrome. Regardless of how it is identified, the importance of identification in PD is necessary to plan the most effective treatment plan. Viewing FS in multiple dimensions also provides a chance to obtain a complete picture of the problem and look at the aging individual from a broader perspective, thus creating more effective preventive and curative measures. It is important to investigate the interactions and determine which one plays a dominant role in the progression of FS in older people.

According to Klietz et al. [2] and Jankowska-Polanska et al. [42], FS can take years to develop, and failure to intervene during this development leads to loss of independence, disability, and death. However, according to Uchmanowicz et al. [20], FS can potentially be slowed down and perhaps even reversed. The key in this regard is early detection of any form of functional impairment at the physical, mental, and social levels. There are a limited number of studies in the literature assessing frailty in PD.

In 2008, Ahmed et al. [43] published a study to determine the prevalence of FS in people with PD; the prevalence of FS was 51.5% in patients with idiopathic PD using the FFI questionnaire. Roland et al. [44] and Özer et al. [45] also obtained similar results in their study. In our study, the prevalence of FS was 95.6%. More studies on the publication market and sharing of experiences will allow FS to be properly diagnosed in PD patients, as there is a risk that exhaustion may be related to changes in the peripheral or CNS in PD patients. Declining physical performance, disturbing gait speed, and increasing depression, which often coexist in PD, are characteristic of the disease but are also determinants of FS. Our results, as well as those of Ahmed et al. [43], indicate that the FS picture may overlap with PD symptoms, and it is necessary to diagnose patients in this direction. The same result was also obtained by He et al. [37] and Wedderburn et al. [46].

Lee et al. [47] cite women’s greater exposure to FS as likely, but found no such relationship in their study; however, they note the need for further research into the relationship between age, gender, and FS. In contrast, a strong correlation with gender was observed in our study. Most frail patients were women (60% *n* = 171), while men predominated in the group without FS (85% *n* = 11).

Brinkman et al. [34] and Lee et al. [47] clearly indicate a reduction in QOL in FS patients. The present study also indicates lower QOL in patients with FS compared to those without FS (*p* < 0.05). However, in our study, the impact of FS on the QOL of PD patients was not confirmed. This issue requires further investigation considering multicenter cooperation and cross-cultural context.

### Study Limitations

There are several potential limitations of this study that should be mentioned. First, all the survey instruments used were of the self-report type, so there is always a risk of bias. Second, the study group could have been more extended and randomly assigned. In addition, this study does not include a control group, which should also be considered in future studies. Another limitation of the present study is the lack of multicenter research on FS in PD patients in cooperation with international partners to include any cross-cultural issues. In order to confirm the results obtained in our study, there is a need to conduct a worldwide study.

## 5. Conclusions

FS is widespread among PD patients. Predictors such as depression, advanced stage of the disease, education, and occupational and economic status significantly affect the QOL level. Our study did not confirm the impact of FS on the QOL level of PD patients, and this study should be continued.

Most PD patients are diagnosed with FS and depression, so the lower QOL of these individuals is also found. Perceiving FS in multiple dimensions also provides an opportunity to obtain a complete picture of the problem and to look at the aging person from a broader perspective and, consequently, provide more effective preventive and curative measures. It is important to study the interactions and determine which of them plays a dominant role in the progression of FS in people with PD. The topics of PD, the co-occurrence of FS with it, and the QOL of people with PD require further scientific research.

## Figures and Tables

**Table 1:** Study group characteristics.

Group *n* = 296								
Variable		$\bar{x}$	Me	Min	Max	Q1	Q3	SD
Age [years]		70.3	69.0	65.0	87.0	65.0	74.0	5.7
PD period [years]		8.2	7.0	1.0	26.0	4.0	10.5	5.6
Variable	Variable category	*n*			%			
Sex	Female	173			58.4			
	Male	123			41.6			
Place of residence	Village	47			15.9			
	City <25 thousand residents	38			12.8			
	City 25–100 thousand residents	39			13.2			
	City >100 thousand residents	172			58.1			
Education	Primary	31			10.5			
	Vocational	59			19.9			
	Secondary	101			34.1			
	Higher	105			35.5			
Marital status	Single	7			2.4			
	Divorced	20			6.7			
	Widowed	71			24.0			
	Single	9			3.0			
	Married	189			63.9			
Occupational activity	Retired	174			58.9			
	Active/working	33			11.1			
	Pension	74			25.0			
	Unemployed	6			2.0			
	Housekeeper	9			3.0			
Material status	Very bad	18			6.1			
	Bad	36			12.2			
	Medium	158			53.3			
	Good	69			23.3			
	Very good	15			5.1			
Household management	By myself	46			15.5			
	I use the help of neighbors, community nurses, and social workers	24			8.1			
	With a close friend	77			26.0			
	With family	149			50.4			
Additional physical activity	No	39			13.2			
	Yes	227			76.7			
	My health condition disables me from exercising on my own	30			10.1			
Increasing knowledge of PD	No	36			12.2			
	Yes	239			80.7			
	My illness prevents me from reading	21			7.1			
Affected social life by PD	Many relationships have been broken	70			23.6			
	Relationships with friends have deteriorated	66			22.3			
	I have no new friends and existing acquaintances have not changed	92			31.1			
	Very favorably, I have expanded my circle of acquaintances	68			23.0			

$\bar{x}$—mean; Me—median; Q1—first quartile; Q3—third quartile; Min—minimum value; Max—maximum value; SD—standard deviation; *n*—number of people; %—percentage of people.

**Table 2:** Information on PD and comorbidities.

Group *n* = 296			
Variable	Variable category	*n*	%
Clinical form of PD	Tremor	41	13.9
	Akinetic-hypertonic	88	29.7
	Mixed	167	56.4
Body site of predominant symptoms	Left	130	43.9
	Right	166	56.1
Comorbidities	Hypertension	147	49.7
	Diabetes	28	9.5
	Asthma	25	8.4
	Kidney failure	9	3.0
	Heart failure	40	13.5
	Cancer	21	7.1
	Osteoarthritis/spinal disease	163	55.1
	Obesity	32	10.8
	Hearing deficiency	46	15.5
	Stroke	12	4.1
	No other diseases	30	10.1

$\bar{x}$—mean; Me—median; Q1—first quartile; Q3—third quartile; Min—minimum value; Max—maximum value; SD—standard deviation; *n*—number of people; %—percentage of people.

**Table 3:** Questionnaire scores of the study group.

	Group *n* = 296							
PDQ-39	Variable	$\bar{x}$	Me	Min	Max	Q1	Q3	SD
	Mobility	46.3	50.0	0.0	97.5	22.5	70.0	27.2
	Activities of daily living	43.3	45.8	0.0	100.0	20.8	62.5	26.6
	Emotional well-being	45.0	41.7	0.0	95.8	25.0	66.7	23.4
	Stigma	35.9	37.5	0.0	93.8	12.5	50.0	25.7
	Social support	27.1	16.7	0.0	100.0	8.3	41.7	24.7
	Cognition	44.8	43.8	0.0	87.5	31.3	62.5	20.9
	Communication	35.2	33.3	0.0	91.7	16.7	50.0	21.9
	Bodily discomfort	52.0	50.0	0.0	100.0	41.7	66.7	22.2
	Total	41.2	42.2	5.2	81.5	26.3	55.1	17.7
TFI	Physical	4.4	5.0	0.0	8.0	3.0	6.0	1.6
	Psychological	3.1	3.0	1.0	4.0	3.0	4.0	0.7
	Social components	1.7	2.0	0.0	3.0	1.0	2.0	0.7
	Total	9.2	9.0	3.0	15.0	8.0	11.0	1.9
SE-ADL		72.9	70	20	100	60	90	17.7
BDI		17.3	16.0	1.0	47.0	8.0	25.0	10.8
UPDRS	Intellectual functioning	4.3	4.0	0.0	14.0	2.0	6.0	3.2
	Activities of daily living	20.7	22.0	2.0	44.0	12.0	27.5	10.1
	Motor skills	22.3	21.0	3.0	52.0	12.0	29.0	12.3
	Complications of treatment	7.1	7.0	0.0	17.0	3.0	10.0	4.5
		***n*** **(%)**						
TFI	FS	*n* = 283 (95.6%)						
	No FS	*n* = 13 (4.4%)						
BDI	No depression	*n* = 89 (30.1%)						
	Moderate depression	*n* = 141 (47.6%)						
	Severe depression	*n* = 66 (22.3%)						

$\bar{x}$—mean; Me—median; Q1—first quartile; Q3—third quartile; Min—minimum value; Max—maximum value; SD—standard deviation.

**Table 4:** Comparisons of PDQ-39 with TFI and BDI.

Variable		PDQ-39—Total							*p* *
		$\bar{x}$	Me	Min	Max	Q1	Q3	SD	
TFI	FS	41.6	42.2	5.2	81.5	26.6	55.1	17.4	0.031
	No FS	32.1	23.2	14.9	78.0	16.7	39.2	21.8	
BDI	No depression (A)	33.0	28.5	5.2	78.0	21.4	42.9	16.8	<0.001
	Moderate depression (B)	41.0	40.8	11.5	80.6	29.4	50.3	15.4	
	Severe depression (C)	52.6	51.4	11.8	81.5	44.3	65.0	17.5	

$\bar{x}$—mean; Me—median; Q1—first quartile; Q3—third quartile; Min—minimum value; Max—maximum value; SD—standard deviation; * Mann–Whitney U-test.

**Table 5:** Comparison of TFI performance with sex, marital status, occupational activity, material status, and mode of household management.

Group *n* = 296						
		TFI				*p* *
		No FS		FS		
		*n*	%	*n*	%	
Sex	Female	2	15.4	171	60.4	0.001
	Male	11	84.6	112	39.6	
Marital status	Single	0	0.0	7	2.5	0.559
	Divorced	0	0.0	20	7.1	
	Widowed	2	15.4	69	24.3	
	Single	0	0.0	9	3.2	
	Married	11	84.6	178	62.9	
Occupational activity	Retired	12	92.3	162	57.2	0.169
	Active/working	0	0.0	33	11.7	
	Pension	1	7.7	73	25.8	
	Unemployed	0	0.0	6	2.1	
	I take care of the house	0	0.0	9	3.2	
Marital status	Very bad	0	0.0	18	6.4	0.296
	Bad	1	7.6	35	12.4	
	Medium	6	46.2	152	53.6	
	Good	6	46.2	63	22.3	
	Very good	0	0.0	15	5.3	
Household management	On my own	2	15.4	44	15.5	0.051
	I use help from neighbors, community nurses, and social workers	0	0.0	24	8.5	
	With a close friend	0	0.0	77	27.2	
	With family	11	84.6	138	48.8	

*n*—number of subjects; %—percentage of subjects; * chi-square test.

**Table 6:** Linear regression analysis evaluating the influence of selected variables on the PDQ-39 total score.

Variable		One-Way Linear Regression Analysis				
		B	SE	t	*p*-Value	ß
Age [years]		−0.12	0.18	−0.67	0.503	−0.04
PD period [years]		0.73	0.18	4.08	<0.001 *	0.23
SE-ADL		−11.75	5.80	−2.02	0.044 *	−0.12
BDI		0.02	0.10	0.24	0.814	0.01
UPDRS	Intellectual functioning	1.93	0.30	6.50	<0.001 *	0.35
	Activities of daily living	0.83	0.09	9.18	<0.001 *	0.47
	Motor skills	0.57	0.08	7.32	<0.001 *	0.39
	Complications of treatment	1.48	0.21	6.91	<0.001 *	0.37
TFI	Physical	−0.09	0.65	−0.13	0.894	−0.01
	Psychological	−0.50	1.44	−0.35	0.729	−0.02
	Social components	−0.78	1.40	−0.55	0.580	−0.03
	Total	−0.24	0.53	−0.45	0.655	−0.03
**Variable**	**Variable category**					
Sex (ref. female)	Male	−1.88	1.04	−1.80	0.072 *	−0.10
Place of residence (ref. village)	City <25 thousand residents	3.79	2.38	1.59	0.112 *	0.11
	City 25–100 thousand residents	0.88	2.36	0.37	0.710	0.03
	City >100 thousand residents	−1.02	1.57	−0.65	0.515	−0.04
Education (ref. primary)	Vocational	1.45	1.96	0.74	0.461	0.04
	Secondary	0.37	1.67	0.22	0.827	0.01
	Higher	−6.29	1.65	−3.80	<0.001 *	−0.22
Marital status (ref. widowed)	Single	−3.82	5.52	−0.69	0.490	−0.10
	Divorced	0.52	3.64	0.14	0.886	0.02
	Single	0.18	4.96	0.04	0.971	0.00
	Married	−1.94	2.23	−0.87	0.386	−0.09
Occupational activity (ref. pension)	Retired	1.45	1.96	0.74	0.461	0.04
	Active/working	0.37	1.67	0.22	0.827	0.01
	Unemployed	−6.29	1.65	−3.80	<0.001 *	−0.22
	Housekeeper	2.32	5.93	0.39	0.697	0.03
Material status (ref. very bad)	Bad	5.59	2.59	2.15	0.032 *	0.13
	Medium	−2.94	1.74	−1.69	0.093 *	−0.10
	Good	−7.97	2.11	−3.78	<0.001 *	−0.23
	Very good	−7.83	3.66	−2.14	0.033 *	−0.15
BDI (ref. no depression)	Moderate depression	−1.16	1.27	−0.91	0.362	−0.06
	Severe depression	10.37	1.53	6.78	<0.001 *	0.42
TFI (ref. no FS)	FS	4.74	2.50	1.90	0.059 *	0.11

B—non-standardized regression coefficient B; SE—standard error; t: B/standard error; ß—standardized regression coefficient ß; * variables included in the multivariate model (criterion: *p* < 0.3 in the one-factor model).

## Data Availability

The data presented in this study are available on request from the corresponding author.
